# A [68Ga]Ga-DOTANOC PET/CT Radiomic Model for Non-Invasive Prediction of Tumour Grade in Pancreatic Neuroendocrine Tumours

**DOI:** 10.3390/diagnostics11050870

**Published:** 2021-05-12

**Authors:** Alessandro Bevilacqua, Diletta Calabrò, Silvia Malavasi, Claudio Ricci, Riccardo Casadei, Davide Campana, Serena Baiocco, Stefano Fanti, Valentina Ambrosini

**Affiliations:** 1 Advanced Research Center for Electronic Systems (ARCES), University of Bologna, I-40125 Bologna, Italy; s.malavasi@unibo.it (S.M.); s.baiocco@unibo.it (S.B.); 2 Department of Computer Science and Engineering (DISI), University of Bologna, I-40136 Bologna, Italy; 3 Department of Nuclear Medicine, DIMES, Alma Mater Studiorum, University of Bologna, I-40126 Bologna, Italy; dilecala@gmail.com (D.C.); stefano.fanti@aosp.bo.it (S.F.);; 4 Research Institute on Global Challenges and Climate Change (Alma Climate), University of Bologna, I-40126 Bologna, Italy; 5 IRCCS Azienda Ospedaliero Universitaria di Bologna, I-40138 Bologna, Italy; claudio.ricci6@unibo.it (C.R.); riccardo.casadei@unibo.it (R.C.); davide.campana@unibo.it (D.C.); 6 Department of Surgery, DIMEC Alma Mater Studiorum, University of Bologna, S.Orsola-Malpighi Hospital, I-40138 Bologna, Italy; 7 NET Team Bologna, ENETS Center of Excellence, I-40138 Bologna, Italy; 8 Department of Oncology, DIMES Alma Mater Studiorum, University of Bologna, S.Orsola-Malpighi Hospital, I-40126 Bologna, Italy

**Keywords:** [68Ga]Ga-DOTANOC, pancreatic neuroendocrine tumour, machine learning, biomarker, standardized uptake value

## Abstract

Predicting grade 1 (G1) and 2 (G2) primary pancreatic neuroendocrine tumour (panNET) is crucial to foresee panNET clinical behaviour. Fifty-one patients with G1-G2 primary panNET demonstrated by pre-surgical [68Ga]Ga-DOTANOC PET/CT and diagnostic conventional imaging were grouped according to the tumour grade assessment method: histology on the whole excised primary lesion (HS) or biopsy (BS). First-order and second-order radiomic features (RFs) were computed from SUV maps for the whole tumour volume on HS. The RFs showing the lowest *p*-values and the highest area under the curve (AUC) were selected. Three radiomic models were assessed: A (trained on HS, validated on BS), B (trained on BS, validated on HS), and C (using the cross-validation on the whole dataset). The second-order normalized homogeneity and entropy was the most effective RFs couple predicting G2 and G1. The best performance was achieved by model A (test AUC = 0.90, sensitivity = 0.88, specificity = 0.89), followed by model C (median test AUC = 0.87, sensitivity = 0.83, specificity = 0.82). Model B performed worse. Using HS to train a radiomic model leads to the best prediction, although a “hybrid” (HS+BS) population performs better than biopsy-only. The non-invasive prediction of panNET grading may be especially useful in lesions not amenable to biopsy while [68Ga]Ga-DOTANOC heterogeneity might recommend FDG PET/CT.

## 1. Introduction

Pancreatic neuroendocrine neoplasia (panNEN) represent approximately 3% of all pancreatic tumours [1]. Although they are generally characterised by a more indolent behaviour as compared to the more aggressive pancreatic adenocarcinoma, panNEN may show different clinical behaviour depending on tumour grade, with grade 1 (G1) and 2 (G2) pancreatic neuroendocrine tumours (panNET) showing more favourable outcome as compared to NET G3 and neuroendocrine carcinoma. Prognosis is also strongly influenced by cells’ differentiation [2].

The possibility to non-invasively assess the primary lesion tumour grade by positron emission tomography/computed tomography (PET/CT)-derived parameters is extremely appealing, potentially overcoming the limits of biopsy sampling, hampered by being both invasive and not always representative of the whole lesion.

To date, most papers have correlated the easily accessible 68Ga-DOTApeptides PET/CT maximum standardised uptake value (SUVmax) of a given lesion with its pathological grade [3,4,5]. However, the SUVmax corresponds to a single voxel measure and therefore it may not take into account potential heterogeneity of the whole lesion. In fact, it is well known that neuroendocrine tumour lesions with varying differentiation grades and somatostatin receptors (SSTR) expression may co-exist within the same patient. From a clinical perspective, the detection of heterogeneity in SSTR expression is crucial as it usually indicates the presence of more aggressive (generally [18F]FDG-positive) tumour foci [6,7]. 

Texture features are a second-order level of image analysis that show promising results in several tumour settings [8,9,10,11]. However, to the best of our knowledge, this is the first report of the use of [68Ga]Ga-DOTANOC PET/CT-derived radiomic features (RFs) to predict primary tumour grade in panNET patients.

The aim of the present study was to determine if image-based RFs extracted from pre-surgical [68Ga]Ga-DOTANOC PET/CT may be used to predict grade in G2 and G1 primary panNET patients.

## 2. Materials and Methods

### 2.1. Patient Population

The population under study consists of a subgroup of patients with panNET selected from two different prospective electronic archives, one collecting cases with different types of pancreatic tumour primaries and eligible for primary tumour resection (Ethical Committee approval: 64/2017/U/Oss on 18 July 2017) and the other prospectively collecting cases of patients with neuroendocrine tumour who had undergone [68Ga]Ga-DOTANOC PET/CT at the Nuclear Medicine Unit of the S. Orsola-Malpighi Hospital of Bologna (Ethical Committee approval: 131/2017/O/Oss on 16 May 2017). All patients gave written consent for their anonymous data to be collected in the respective electronic archive. Collected clinical data included gender, age, tumour grade, PET/CT, and conventional imaging results.

The current analysis included patients presenting (a) a histologically confirmed G1-G2 panNET, (b) a [68Ga]Ga-DOTANOC PET/CT performed before surgery showing uptake in the primary panNET lesion, and (c) a staging diagnostic conventional procedure (CT or EUS) showing the primary pancreatic lesion. Patients were further divided into two subgroups according to the pathological assessment method: histology on the whole excised primary lesion (HS) or biopsy of the primary lesion (BS).

WHO 2019 [12] classification was used for pathological grading, according to Ki-67 level and cells morphology [13].

### 2.2. Image Acquisition and Analysis

[68Ga]Ga-DOTANOC PET/CT was performed following standard European Association of Nuclear Medicine (EANM) procedures [14]. [68Ga]Ga-DOTANOC was synthesized at the Radiopharmacy of the Nuclear Medicine Unit of S. Orsola–Malpighi Hospital. [68Ga] was eluted from a [68Ge]/[68Ga] generator, and DOTANOC was labelled with 68Ga [15]. [68Ga]Ga-DOTANOC PET scans were obtained using a dedicated PET/CT scanner (Discovery STE; GE Healthcare, Chicago, IL, USA). PET emission images were recorded for 3 min per bed position; CT images were used for non-uniform attenuation correction (acquisition parameters, 100 kV, 120 mA, 0.6 s, 3.75 mm). All images were corrected for scatter, randoms, dead time, and decay. Images were reconstructed with a 2-dimensional ordered-subset expectation maximization iterative algorithm (2 iterations, 28 subsets). Any area with an intensity higher than the background was considered positive for SSTR expression. SUVmax was calculated using a volume of interest including the whole primary lesion. For the purpose of the following texture analysis, when more than one lesion was present in the pancreas, the one with the highest uptake was segmented.

### 2.3. Radiomic Model

**Figure 1:**
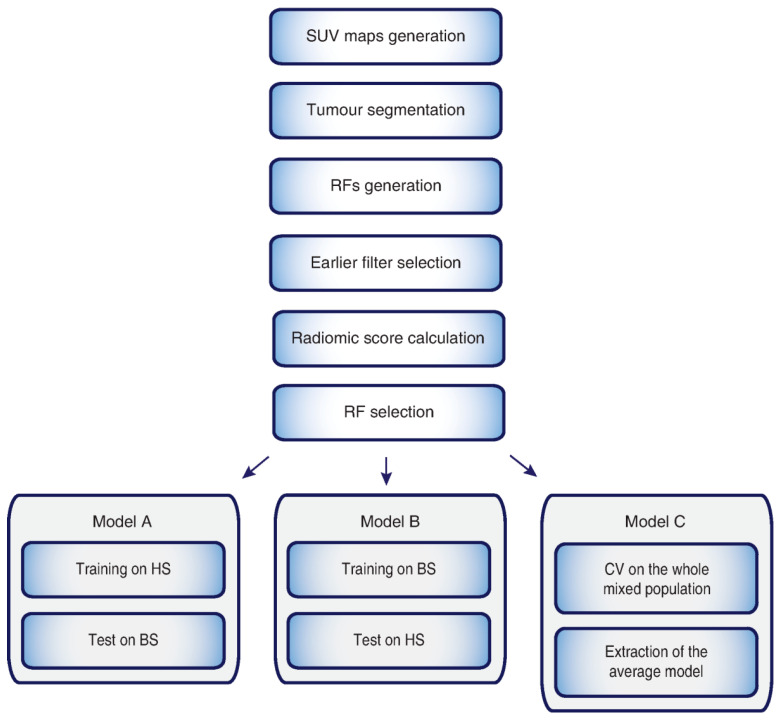
Workflow employed to extract radiomic features (RFs). First, segmentation of the primary lesion was performed on the transaxial PET images, then RFs were generated and extracted. The single and coupled RFs showing the lowest *p*-values and the highest AUC were selected and employed to develop the three predictive radiomic models used in this study. In model C, cross-validation (CV) on the whole mixed population was performed. Tumour grade was assessed either by histology on the whole excised primary lesion (HS) or on its biopsy (BS).

One of the key issues in machine learning to train a supervised classification procedure is the availability of a reliable ground-truth, here represented by the tumour lesion’s grade. Although grade assessment based on either biopsy samples or whole surgical specimens is considered equivalent for clinical management in most cases, HS and BS discrepancies can be encountered in clinical practice (since the BS may be biased by sampling error, especially in heterogeneous or large lesions). Since the aim of this paper is to train a classifier with the highest certainty degree, HS is to be considered the most reliable “ground truth”, although it is beyond dispute that most of BS grading matches with HS ones. Three different predictive radiomic models were developed and compared (Figure 1): (A) the first, trained on HS and validated on BS; (B) the second, trained on BS and validated on HS; and (C) the third, trained and validated using the whole dataset, with all patients mixed up, using the cross-validation (CV) technique, normally employed to cope with the so-called selection bias. 

All the models were built using a linear classifier, that is, the Linear Discriminant Analysis (LDA) [16]. It is worth noting that the features used in all models were extracted and selected once from HS patients only (expectedly the most reliable “ground truth”), during training of model A. For the same reason, the reproducibility analysis was carried out using the training stage of model A. The procedures adopted to compute the three models can be resumed through the following three steps.

(1) **Segmentation** (performed on the whole dataset). Segmentation of whole tumour volumes was performed manually on transaxial PET images by the nuclear medicine physician using ImageJ (https://imagej.nih.gov/ij/, accessed on 5 April 2021 [17]), on regions of interest (ROIs) thresholded at 40% SUVmax value.

(2) **Feature generation** (performed on HS only). On these SUV maps, four symmetric grey-level co-occurrence matrices (GLCMs) quantized at 64 grey-levels were first generated considering four distances d = {1, 2, 3, 4}, then averaged over four angles θ = {0°, 45°, 90°, 135°}. Finally, 12 first-order RFs (Supplementary Material Table S1) were directly computed on the SUV maps and 12 s-order RFs (Supplementary Material Table S2) on each GLCM, yielding 48 s-order RFs (12 for each d), for a total of 60 RFs. All RFs were normalized. 

(3) **Feature selection** (performed on HS only). To prevent overfitting, we focused our attention separately on the 60 single RFs and on all the 1770 RF couples (more details in Supplemental Data). First, we performed an earlier filter selection based on RF correlation values, so that remaining single RFs were not highly correlated and remaining RF couples had a very low mutual correlation with each other (more details in Supplemental Data). Then, the best cut-off provided by the Youden Index (YI) computed on the ROC curve was selected for single RFs, while for each RF couple the LDA was applied to extract the radiomic signature (RAD) [18]. The radiomic score (RS) was thereafter computed for each patient for all the single and coupled RFs. Subsequent RF selection was carried out as follows: (1) the capability of RFs to separate G2 and G1 patients based on their RS was assessed through a statistical test, filtering RFs with non-significant *p*-values; (2) the AUC was computed to assess the discrimination power of the RF surviving step (1). Finally, single and coupled RFs showing the lowest *p*-values and the highest AUC were selected. As regards the reproducibility of results, it was assessed by perturbing the original segmentation through binary dilation and erosion procedures [19], so to allow computing the selected features, and the radiomic models, on different (greater or smaller) volumes. The procedure was conceived to obtain for each patient the same percentage changes (nominally, from 1% to 100%, with step 1%).

**Figure 2:**
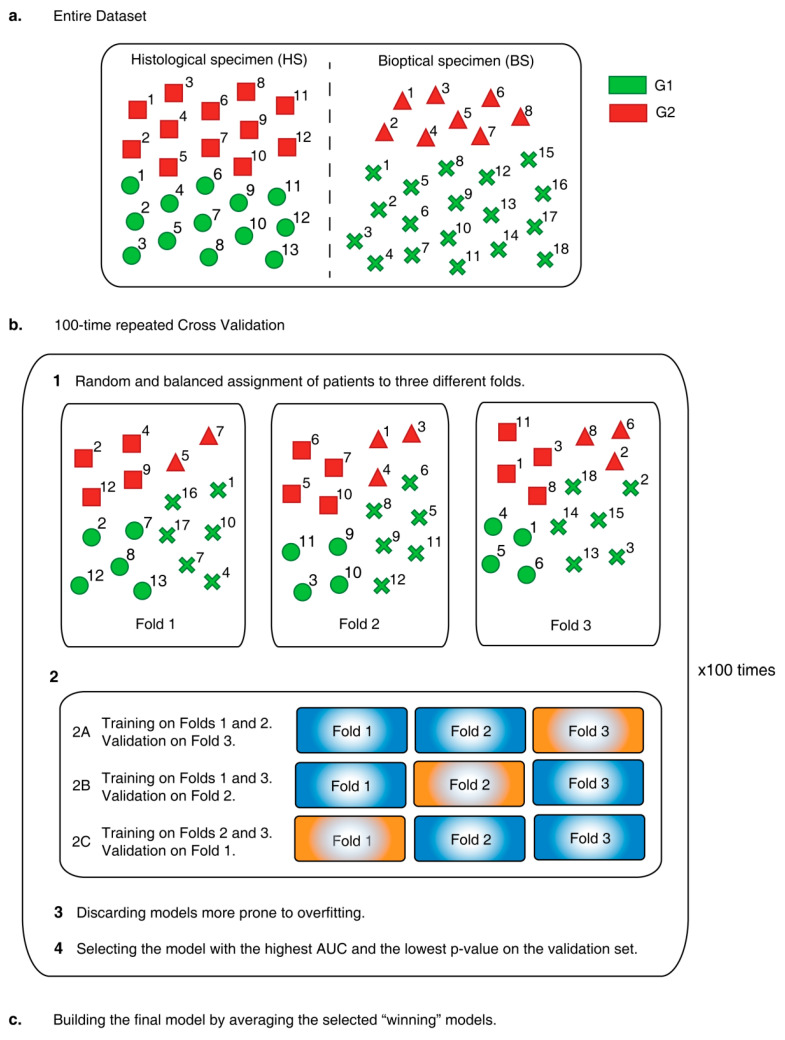
The procedure adopted to build model C starting from a “mixed” population (**a**) of patients with grade 1 (G1) and 2 (G2) pancreatic neuroendocrine neoplasia assessed through the analysis of histological (HS) or biopsy (BS) samples is described. In particular, repeated cross validation is applied 100 times to extract as many radiomic models trained and tested on randomly selected and balanced groups of patients (**b**) and exploited to build the final radiomic model (**c**).

**Cross Validation (CV)**. The CV adopted to train and validate model C is a 100-times repeated 3-fold CV (Figure 2).

Here, the whole dataset was split into three non-overlapping folds where, in each round, one fold was used in turn for validation and the remaining two for training, thus yielding three runs for each of the 100 repetition. Each fold was randomly populated with G1 and G2 patients, coming from HS and BS datasets, according to the stratified sampling paradigm, that is, keeping both the ratios HS/BS and G1/G2 constant to reflect the proportions in the whole dataset. For each run, a new radiomic model was trained and validated; ROC and AUC were computed separately on the training and validation sets and the prediction performance was measured using the AUC. In particular, in each run the models more prone to overfitting (i.e., with AUC greater for validation than for training set) were discarded and the remaining model yielding the highest AUC in the validation set was selected as the “winner”. At the end, at most 100 trained and 100 validated radiomic models survived. The ultimate predictive radiomic model was achieved by averaging the surviving winning LDA models of the validation sets ([20,21]) (more details in Supplemental Data).

As common in many studies [20,21,22], RFs computation and selection, and model setup were performed using an in-house software based on MATLAB^®^ (MathWorks, Natick, MA, USA).

### 2.4. Statistical Analysis

Pearson correlation coefficient was used for earlier filter selection. The two-tail Wilcoxon rank-sum test was performed to assess the statistical significance (*p*-value ≤ 0.05) of the separability between G2 and G1 for SUVmax values, and for the single and coupled RFs, where the statistical significance was updated according to Holm-Bonferroni correction. Sensitivity, specificity, informedness, positive predictive value (PPV), negative predictive value (NPV), and accuracy were calculated to quantify and compare the discrimination capability of RFs and SUVmax. Statistical analysis was performed on MATLAB^®^. As regards reproducibility, several radiomic models were generated by extracting the selected RFs on differently segmented volumes. Besides assessing the reproducibility of features through the Intraclass correlation coefficient (ICC), we also analysed the reproducibility of the radiomic models by comparing the *p*-values of discrimination, sensitivity, specificity, accuracy, AUC and, importantly, checking whether the mis-discriminated patients changed. 

## 3. Results

### 3.1. Patients’ Population

Overall, 58 patients met the inclusion criteria (HS cohort: *n* = 25 patients; BS cohort: *n* = 33). Seven patients of the BS group were subsequently excluded from texture analysis since primary lesions’ size were too small to generate a GLCM of 64 grey levels. In the final analysis, 51 patients were included in the study (HS group: *n* = 25 patients; BS: *n* = 26; ki67 median [range]: G1 = 1.6 [0.6–2.9] and G2 = 4.0 [3.0–18.6]).

Epidemiological and clinical patients’ characteristics for both cohorts are shown in Table 1.

In the HS groups, tumours presented mostly as a single lesion (17 vs. 8 cases showing multiple lesions within the pancreas). When more than one lesion was present, the one with the highest uptake was segmented. At pathological evaluation, most primary pancreatic lesions were located in the tail (9), followed by the head (7), the body (6), and in two cases the tumour was diffuse while in one case it was in the isthmus. All HS patients underwent surgical excision of the primary tumour and in 14/25 an additional biopsy pre-surgical sampling was also performed: pre-surgical BS identified 6 G1-cases and 8 G2-cases, while HS confirmed BS results in 10/14 patients (the remaining four discordant cases were one case upgraded from G1 to G2 and three cases downgraded from G2 to G1), this yet further supporting the choice of separating BS and HS. In patients presenting multiple lesions within the pancreas, the pathological grade was the same in all lesions.

In the BS group, primary tumours were mostly located in the head (14), followed by body (5), tail (4), and isthmus (2). Only one patient showed multiple primary lesions (body and tail). Biopsy sampling identified 18 G1-cases and 8 G2-cases. In the single case with more than one pancreatic lesion, the pathological grade was the same in all lesions.

### 3.2. Imaging Studies

Diagnostic conventional imaging data were available in all patients and detected the presence of the primary panNET in all cases (HS group: mean lesion dimensions 27.8 mm [range 8–85 mm; median 23.5 mm]; G1 vs. G2 mean lesion dimension: 19.8 ± 11.9 mm vs. 35.8 ± 20.3 mm; BS group: mean lesion dimensions 22.9 mm [range 6–50 mm; median 23 mm]; G1 vs. G2 mean lesion dimension: 19 ± 11 vs. 30 ± 9.2 mm).

**Figure 3:**
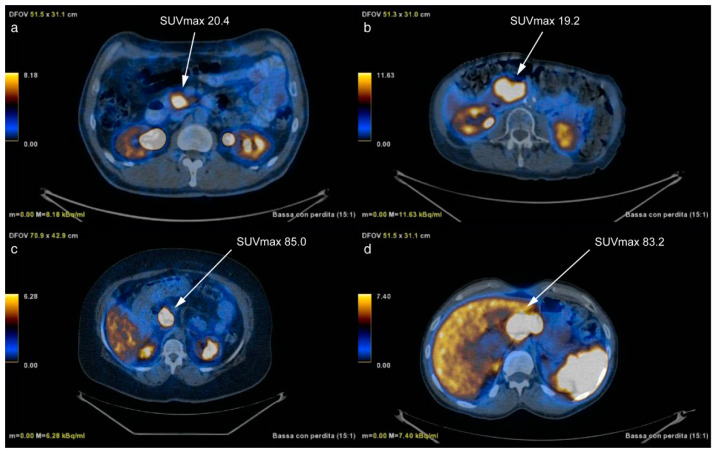
[68Ga]Ga-DOTANOC PET/CT transaxial fused images of two pairs of patients presenting similar SUVmax values of the primary lesion but different tumour grades. In particular, although SUVmax of (**a**,**b**) is similar, (**a**) is G1 while (**b**) is G2; accordingly, (**c**,**d**) present similar SUVmax primary tumor values but (**c**) is G1 while (**d**) is G2. On the contrary, the primary lesion’s grade was correctly identified (G1: **a**,**c**; G2: **b**,**d**) when using the selected RFs.

HS group primary lesion’s SUVmax values did not significantly differ between G1 (36.9 ± 23.5, [6.9–84.8]) and G2 (45.3 ± 28.6, [15.0–95.7]) primary lesions (*p*-value = 0.60), with AUC = 0.56 (95% CI, 0.33–0.77), near to the threshold of random assignment (sensitivity = 42% and specificity = 85%, at Youden cut-off = 0.27). This means that using the SUVmax only 5/12 G2 were detected, leading to PPV = 71%, NPV = 61% and accuracy = 64%. Figure 3 shows two sets of patients presenting comparable, almost identical, SUVmax values corresponding, however, to different lesions’ grades.

On the contrary, the primary lesion’s grade was correctly identified when using the selected RFs that allowed predicting G2 (b, d) and G1 (a, c) cases. Since SUVmax proved not to be able to discriminate G1 from G2 patients, it was not further evaluated.

### 3.3. RF Selection

**Figure 4:**
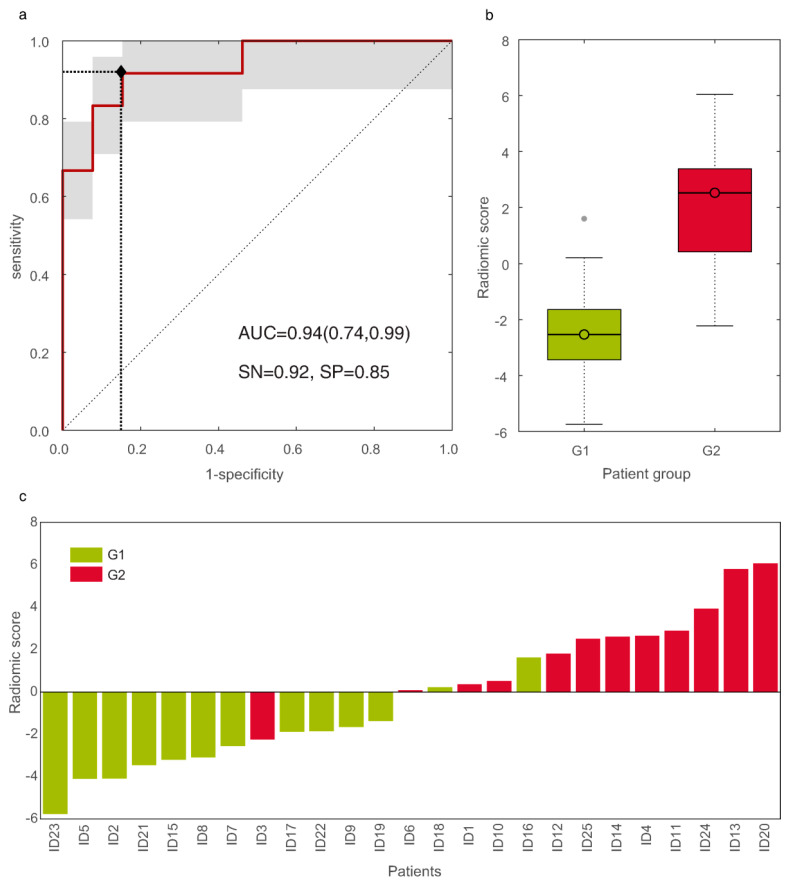
The ROC curve of the discriminative radiomic model (**a**). Boxplots of radiomic scores (RS) of grade 1 (G1) and grade 2 (G2) groups, with median values of −2.53 and 2.53, respectively (**b**). Waterfall plot of the RS of G1 (green bars) and G2 (red bars) pancreatic neuroendocrine neoplasia (panNET) patients (**c**).

Two single and six coupled RFs derived from SUV maps survived after step (3), all showing accuracy between 0.84 and 0.88. To discriminate G2 from G1 panNET patients, the best result (*p*-value = 0.0002) was provided by the uncorrelated (|ρ| = 0.03) coupled RF (NH_GLCM-2_, E_GLCM-4_), referring to the second-order normalized homogeneity and entropy, respectively, with AUC = 0.94 (95% CI, 0.74–0.99) and ROC shown in Figure 4a.

**Figure 5:**
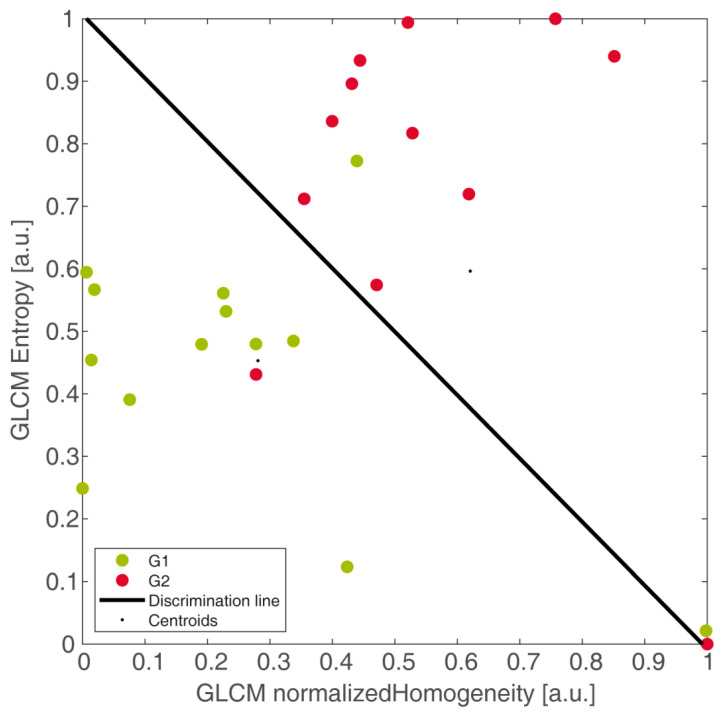
Discrimination of grade 2 (G2, in red) and grade 1 (G1, in green) pancreatic neuroendocrine neoplasia (panNET) patients by second-order normalized homogeneity and entropy, linearly separated by the linear discriminant analysis boundary (in black). Here, patients with grading assessed through histology on the whole excised primary lesion (HS) were well discriminated. This also represents the output of the training stage of model A.

Eleven out of twelve G2 and 11/13 G1 were correctly detected, yielding sensitivity = 92%, specificity = 85%, informedness = 0.76, PPV = 85%, and NPV = 92%, with respect to G2. Boxplots of RS of G1 and G2 groups were reported in Figure 4b. Figure 4c reports the waterfall plot of the RS of G1 (light green bars) and G2 (red bars) groups, with cut-off arising from LDA. In particular, one false negative (FN) (ID3) and two false positive (FP) (ID16 and ID18) were reported, with false discovery rate (FDR) = 0.15. Finally, Figure 5 showed the two groups linearly separated by the LDA, yielding the RAD_A_ (that is, RAD of model A) in Equation (1):

(1) $$RAD_{A}=7.69\;NH_{GLCM-2}+7.58\;E_{GLCM-4}-7.63$$

The linear decision boundary (in black) was in correspondence with RAD_A_ = 0 and represented the optimal separation of patients, the line where the two groups are equally probable. In particular, the top-right part of the plot, where G2 lies, is expressed by the inequality in Equation (2):

(2) $$7.69\;NH_{GLCM-2}+7.58\;E_{GLCM-4}-7.63>0$$

while the bottom-left part where G1 lies is expressed by the inequality in Equation (3):

(3) $$7.69\;NH_{GLCM-2}+7.58\;E_{GLCM-4}-7.63<0$$

Second-order features computed at different distances are well-known to be often highly correlated. This is what happens with d = 1 and d = 2 for NH_GLCM_ (ρ = 0.90) and with d = 3 and d = 4 for E_GLCM_ (ρ = 0.98), whose discrimination performance is reported in Supplementary Material Table S3. Although *p*-values are generally a little worse, they all show very good results, with all AUC greater than 0.90, and same YI = 0.77. 

As regards the single features, it is worth noting that the first ranked is the second-order normalized homogeneity (NH_GLCM-1_, *p*-value = 0.0021), AUC = 0.87 (95% CI, 0.58–1), sensitivity = 92%, specificity = 85% at Youden cut-off (YI = 0.77), followed by first-order entropy (ESUV, *p*-value = 0.0028), AUC = 0.85 (95% CI, 0.58–0.99), sensitivity = 85%, and specificity = 92% at YI = 0.77.

As far as the reproducibility of (NH_GLCM-2_, E_GLCM-4_) is concerned, this coupled RFs allowed ROIs reduction (r) of up to 35%. The separation between G2 and G1 kept statistical significance (*p*-value = 0.0004), with same accuracy = 88% and AUC ranging from 0.92 to 0.94. This yields ICC ranging from 0.98 to 1. Small reductions (r ≤ 8%) showed same mis-discriminated patients: ID3 (FN) and ID16, ID18 (FP). For r > 8%, sensitivity = 83%, specificity = 92%, and ID18 was “replaced” by ID6—they had originally very similar RS, both lying nearby the separating boundary (Figure 4c). As regards dilated volumes, to keep ICC ≥ 0.90, volume changes were to be no greater than 25%. The discrimination between G2 and G1, possible with a lower, still highly significant, *p*-value = 0.002, showed AUC ranging from 0.85 to 0.93. Nonetheless, sensitivity = 92% and specificity = 85% are the same as the original segmentation and, most importantly, the mis-discriminated patients remained the same as well. 

In practice, selecting the best RFs represented the training stage of model A. In consideration of the better performance of the coupled RF (NH_GLCM-2_, E_GLCM-4_) with respect to the single RF (NH_GLCM-1_), the selected couple was chosen to develop all the models.

### 3.4. Predictive Radiomic Models

**Figure 6:**
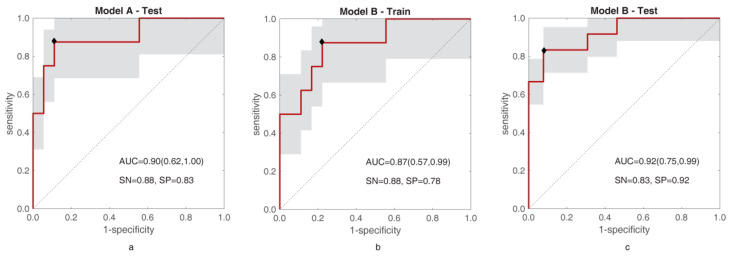
ROC curves for test of model A (**a**), and training (**b**) and test (**c**) of model B are shown. In each figure, the area under the ROC curve (AUC) is reported together with sensitivity (SN) and specificity (SP).

Figure 6 reports the ROC curves of training of model A and training and test of model B, respectively. 

As regards model A, the LDA found selecting the best RF couple was tested on BS yielding AUC = 0.90 (95% CI, 0.62–1.00), sensitivity = 88%, specificity = 83%, informedness = 0.71, PPV = 0.70%, NPV = 94%, accuracy = 0.85, and separates G1-G2 with *p*-value = 0.0035. Instead, using BS to train model B leads to the radiomic signature in Equation (4) (RAD_B_):

(4) $$RAD_{B}=5.66\;NH_{GLCM-2}+4.64\;E_{GLCM-4}-6.80$$

with AUC = 0.87 (95% CI, 0.57–0.99), sensitivity = 63%, specificity = 89%, informedness = 0.51, PPV = 71%, NPV = 84%, accuracy = 81% and a separation with *p*-value = 0.0035. Testing model B on HS leads to AUC = 0.92 (95% CI, 0.75–0.99), sensitivity = 83%, specificity = 92%, informedness = 0.76, PPV = 91%, NPV = 86%, accuracy = 88%, and a separating *p*-value = 0.0002. Supplementary Material Table S4 resumes the main performance parameters of models A and B. As far as model C is concerned, almost all “winning” training models (99/100) did not bring overfitting and they were all statistically significant (*p*-value < 0.0002) in separating G1 and G2, with median (and mean) AUC = 0.93, IQR = 0.03, median sensitivity = 0.85, and median specificity = 0.90. Supplementary Material Table S5 resumes the values of AUC for training and validation referring to all 300 runs, to all non-overfitting models, and to the winning models. As for the “winning” test models, 79 out of 100 were not overfitting and yielded statistically significant G1-G2 separations (*p*-value = 0.003 ÷ 0.048), with median (and mean) AUC = 0.87, IQR = 0.05, median sensitivity = 0.83 and specificity = 0.82, and informedness = 0.76. The average of the 79 radiomic models yields the radiomic signature in Equation (5):

(5) $$RAD_{C}=9.43\;NH_{GLCM-2}+8.16\;E_{GLCM-4}-10.51$$

## 4. Discussion

Although preliminary, our data indicate that [68Ga]Ga-DOTANOC PET/CT-derived RFs can be used to non-invasively predict G2 and G1 panNET primary lesions. In particular, the best RFs were the couple made of GLCM normalized homogeneity at d = 2 and GLCM entropy at d = 4. This highlights the presence of tissues characterized by a locally homogeneous uptake and by heterogeneous uptakes at farther distances, where both features were higher in G2 than in G1. In particular, G2 was mostly associated with a high entropy and a medium normalized homogeneity, whereas G1 showed a medium entropy together with a lower local normalized homogeneity. In practice, the first feature captures a “visible” characteristic of the SUV value distribution, indicating local homogeneity (at distance d = {1,2}), while the entropy encodes the “latent” heterogeneity (at distance d = {3,4}) of tumour tissue and, together, the characteristics of tumour habitat. As regards the reproducibility, the selected RFs have shown to be highly tolerant to changes in volume segmentation, although more with reduction than with dilation. On the other hand, this is expected for two reasons. First, enlarging the volumes tends to include more regions potentially different from the tumour. Second, with an equal percentage of change, volume dilation involves a much higher number of voxels. Nonetheless, the radiomic model built on the dilated segmentations made the same mis-discriminations, with the same patients, as the original model. 

The main challenge of this study was having patients whose grade was assessed with either histological sample or biopsy. These two sampling methods, although considered clinically equivalent, may theoretically harbour a different certainty degree, which could mislead the supervised training stage of a predictive model. Models A and B were conceived to try understanding the effects of using BS as the ground truth to develop a predictive radiomic model, in line with clinical practice. Comparing the training performance of both models, model A trained on HS was the best for all parameters, including AUC (0.94), while model B trained on BS showed a smaller AUC (0.87), with a wider CI. What is more interesting is the behaviour of the models in the test, where model B applied to HS showed an AUC (0.92) greater than in training (AUC = 0.87). While overfitting was usually considered among the first causes, here this can be explained with BS being less representative of the whole lesion. In fact, when training model A (based on HS), G2 among the BS group was correctly predicted (just one more FP than in training). This discrepancy of behaviour between models A and B prompted us to explore the possibility to achieve a good predictive model employing the entire population, with a “hybrid” ground-truth, using cross-validation, routinely chosen to face the effects of selection bias in predictive models. The average performance of model C is still very good in terms of median AUC (0.86), sensibility (0.83), and specificity (0.82), although overall lower than model A. In practice, although model A is the best one, it should be cross-validated on a wider HS dataset to confirm its performance. Nevertheless, the performance of model C, trained also using biopsy ground truth, is probably biased towards low values. Therefore, the expected performances of the best radiomic model are reasonably in between those of models C and A.

It can be argued that in the clinical setting, biopsy results are considered reliable to assess pathological grade in patients not amenable to surgical excision of the whole mass. However, even if the concordance rate of 2019 WHO [12] classification between endoscopic ultrasound-guided fine needle aspiration and biopsy (EUS FNA/FNB) and surgical specimen [23] is high (87.5%) in tumours <20 mm, it is much lower (57.1%) in tumours ≥20 mm. Considering that in the BS, mean and median lesions’ dimension were both >20 mm, the lower sensitivity rate obtained in this subgroup may be more likely related to the biopsy sampling error rather than an effective incompetence of the radiomic model.

It is well known that the disease outcome is strongly influenced by tumour grade [2], usually assessed using an invasive approach such EUS FNA/FNB. Being a whole-body procedure, PET/CT allows the characterisation of the whole tumour burden, provides SSTR expression of all lesions, and is recommend for disease staging. However, the most attractive and easy-to-use PET/CT-derived parameter (SUVmax), defined as the hottest voxel within a volume of interest, represents a single-pixel measurement. Although commonly used in clinical practise, it may be affected by the amount of image noise [24]. A correlation between SUVmax values and SSTR expression [4,5] was reported, but no reference values were indicated to differentiate G1 from G2 tumours. However, our results showed that SUVmax is not effective in discriminating G1 and G2 tumours. 

The distinction between the two grades is clinically relevant as G1 tumours generally show an indolent behaviour while G2 tumours may present varying outcomes, since this group includes tumours with a wider range of Ki-67 values (from 3 to 20). In particular, the possibility to non-invasively evaluate tumour grade at staging may be particularly relevant in small panNETs (<20 mm) and especially for those of 1–2 cm in diameter. For non-functioning lesions <1 cm, it is widely accepted that a “wait and see” approach is sufficient, regardless of tumour grading, as recommended by current ENETS guidelines [25]. The same cannot be said for lesions of 1–2 cm: In fact, several authors dispute the decision of surgical intervention based exclusively on dimensional criteria and remark the importance of grading assessment. Paik et al. [26] conducted a multicentre study on 158 patients with small panNETs ≤2 cm at baseline imaging, excluding G3 and functional panNETs, and found that the WHO classification G2 was the only predictive factor of malignant potential according to univariate and multivariable analysis (HR 13.97, 95% CI 2.60–75.03, *p* = 0.002). Milione et al. [27] found that Ki-67 was one of the major prognostic factors in panNEN and suggested that pre-surgical evaluation of Ki-67 should be included in the algorithm guiding clinical decision on resection of panNEN of 1–2 cm in size. Given the small size of our most reliable samples (HS), we did not consider it appropriate to perform a subanalysis subdiving patients on the basis of lesions’ dimensions but, given the clinical implications, it will certainly be included in future studies on a larger population.

Moreover, in clinical practice, biopsy is often performed on a distant metastasis but, as Grillo et al. stated [28], NETs frequently show differences in grade between primary sites and their synchronous/metachronous metastases, and the assessment of Ki-67 at all sites may prove to be significant for patient management. A non-invasive method to assess grade may contribute to characterise the primary tumour, avoiding its pathological assessment. 

However, it should be outlined that the commonly used invasive methods, such as EUS or CT-guided tissue biopsy, are not feasible in cases of difficult-to-reach lesions or might provide a sample of an area that is not representative of the whole tumour lesion [29]. Another important point to keep in mind is that tumour heterogeneity may hamper accurate grading in panNEN [30,31].

Radiomics is a deeper approach to image analysis, beyond routine visual interpretation. Although very preliminary, published results on the use of radiomics in other tumour settings (mostly lung primary lesions) seem promising [8,9]. Radiomics offers the possibility to analyse the distribution of the tracer in a given lesion, increasing the information that can be derived from routinely acquired standard PET images [32]. In fact, by analysing the distribution and relationship of pixel or voxel grey levels in the image, radiomics may provide an estimate of tumour heterogeneity [33]. The value of CT/MR radiomic features to predict the neuroendocrine lesion grade has been recently published [34,35,36,37]. RF exhibited statistical significance among 77 patients with panNET, allowing the discrimination of G1 from G2/G3 lesions [35]. Canellas et al. reported that the odds of a tumour with high entropy values being an intermediate- or high-grade pancreatic neuroendocrine tumour were 3.7 times as high as those in tumours with low entropy values in CT radiomics [38]. Nonetheless, the employment of radiomics in clinical practise is still experimental, since there is not extensive evidence supporting its utility or reproducibility. In fact, several papers indicate an apparent lack of reproducibility of radiomic studies, mostly related to the different methods used for lesions’ segmentation and features extraction and of the difficulties in employing the derived radiomic results in different clinical settings (for example, implementation in different tomographs at different centres). Moreover, several methods have been described for tumour delineation (manual, thresholding, stochastic, or learning based), however, there is no evidence that one is more accurate than the others [39].

To our knowledge, this is the first paper correlating [68Ga]Ga-DOTANOC PET/CT RFs to the corresponding panNET primary tumour grade. Considering the strong link between the radiopharmaceutical uptake and the available treatment options targeted to the SSTR expression (hot or cold somatostatin analogues), radiomics seems to be particularly promising in the evaluation of NET patients: the presence of high [68Ga]Ga-DOTANOC heterogeneity might correspond to areas of lower differentiation, which are less likely to respond to target treatment and are associated to a poorer prognosis. FDG PET/CT is considered accurate to assess the presence of undifferentiated clones, however, it is not routinely performed in all cases, especially in low-grade tumours. In fact, another open issue in neuroendocrine tumour management is the question of whether to routinely employ [18F]FDG to identify the presence of undifferentiated clones [40]. It can be speculated that the detection of high [68Ga]Ga-DOTANOC heterogeneity might indicate the need of an additional [18F]FDG PET/CT.

Among the limitations of the study, one of the most relevant is certainly the small population, mostly due to the difficulty of finding patients with this rare disorder that had a pathologically confirmed disease on the primary lesion (and not merely on a secondary one) with a pre-surgical [68Ga]Ga-DOTANOC PET/CT, which are both indispensable conditions to train the radiomic model. 

## 5. Conclusions

RFs coupling local homogeneity and farther heterogeneity of SUV values are able to accurately predict G2 and G1 primary panNET. Since visual analysis and SUVmax assessment are not accurate for grade prediction, a radiomic model may provide a non-invasive tool to discriminate G2 from G1 tumours especially in selected patients’ groups (e.g., those not amenable to biopsy). In fact, the radiomic model can be implemented in the same computer where images are reviewed and, following segmentation, it could be employed to assess the specific RF of a panNET primary lesion, in an accessible way almost as the measure of the SUVmax in a given ROI. Differentiating G2 from G1 tumours might also support the surgeon in the therapeutic choice (e.g., follow-up versus resection) in small lesions (<2 cm), especially in those >1 cm, avoiding an invasive procedure such as FNB-biopsy. Finally, the detection of [68Ga]Ga-DOTANOC heterogeneity might strengthen the indication to [18F]FDG PET/CT even in well differentiated NET patients.

From our model analysis, we can state that (i) the best choice is to use a model trained only on patients with histological ground truth while (ii) biopsy grading seems to worsen the training stage and the performance of a predictive radiomic model. Nevertheless, (iii) using a population with a “hybrid” ground truth (in line with clinical practice), the average model achieved through CV makes the prediction performance very good indeed.

## Figures and Tables

**Table 1:** Clinical and epidemiological patients’ data in the two cohorts, HS (histology sample) and BS (biopsy sample).

	HS	BS
Baseline Characteristics	Total Patients *n* = 25 (%)	Total Patients *n* = 26 (%)
Sex		
M	11 (44)	13 (50)
F	14 (56)	13 (50)
Age		
≤65 years	18 (72)	16 (62)
>65 years	7 (28)	10 (38)
Tumour site		
Head	7 (28)	14 (54)
Body	6 (24)	5 (19)
Tail	9 (36)	4 (15)
Isthmus	1 (4)	2 (8)
Diffuse	2 (8)	1 (4)
Tumour Size (median, 95 CI) [mm]	27.8 (8 to 85)	22.9 (6 to 50)

## Data Availability

The data is not available because of patients’ privacy.

## Supplementary Materials

The following are available online at https://www.mdpi.com/article/10.3390/diagnostics11050870/s1, Table S1: First-order RFs computed in the ROI, with related formulas and description; Table S2: Second-order RFs computed in the ROI, with related formulas and description; Table S3: Main performance of the coupled features (NH_GLCM-{1,2}_, E_GLCM-{3,4}_); Table S4: Resuming the main performance parameters of models A and B on training (HS) and validation (BS) cohorts. ρ = Pearson correlation coefficient, SN = sensitivity, SP = specificity, ACC = accuracy, PPV = positive predictive value, NPV = negative predictive value, I = informedness, AUC = area under ROC curve; Table S5: Resuming AUC values for training and validation of model C referring to all 300 runs, to all non-overfitting models, and to the winning models found. N = number of runs, Std = standard deviation, Med = median, IQR = interquartile range.
