# Supplementary material for: A [68Ga]Ga-DOTANOC PET/CT Radiomic Model for Non-Invasive Prediction of Tumour Grade in Pancreatic Neuroendocrine Tumours
Source: Diagnostics (Basel). 2021 May 12;11(5):870. doi: 10.3390/diagnostics11050870 (PMC8150289; doi:10.3390/diagnostics11050870)
Supplement: Supplementary file 1 [file diagnostics-11-00870-s001.zip › diagnostics-1217162-SI.pdf]

SOME DEFINITIONS

RF: Radiomic feature
ROI: Region of interest (here, the whole pancreas lesion)
N_p: The number of voxels of the ROI
N_g: the number of discrete SUV intensity values inside the ROI
GLCM: Gray level co-occurrence matrices
$p(i)$: the probability of the *i* pixel value (*i.e.,* the number of voxels with value *i*, normalized by N_p)

RADIOMIC FEATURES: FORMULAS

First and second order RFs are described by an acronym of the feature, followed by the kind of data which the feature is calculated on. As an example, in the first order features the standard deviation (SD) calculated on SUV voxels in the ROI (or map) is described by the acronym SD_{SUV}.

First order features

12 statistical descriptors were computed on the set of N_p SUV voxels in the ROI.

Second order features

GLCMs were generated on SUV voxels of the ROIs. In particular, the conditional joint probabilities $p((i,j)|(d,\vartheta))$ with which two pixels having relative polar coordinates (d,ϑ) appear with intensities (i,j) were computed for four ϑ angles, $0, \frac{\pi}{4}, \frac{\pi}{2}, \frac{3}{4}\pi$, d varying between 1 and 4. Then, for each d, a resuming GLCM $p((i,j)|d,\cdot)$ was calculated as the average of the GLCM computed for the different ϑ. On those GLCM 12 radiomic feature were computed. The acronyms are similar to those used for the first order features with the addition of d value. For instance, the Normalized Homogeneity (NH) computed on GLCM with distance d = 4 pixels is pointed out as $NH_{GLCM\text{-}4}$.

FEATURE SELECTION: Earlier filter selection

After generating RFs, we performed an earlier filter selection to reduce the number of highly or moderately correlated single and coupled RFs to prevent redundant results. To this purpose, a four-step procedure was followed:

1) RFs pre-processing: the Pearson correlation coefficient (ρ) was calculated for each RFs combination.
2) Filtering highly correlated RFs: RFs showing a very high correlation (ρ>0.85) with other RFs were discarded from both the single and the coupled RFs to be analysed. Finally, 15 single and 105 coupled RFs remained.
3) Filtering the coupled RFs survived at step 2): since these are highly correlated features, here we can afford to use a lower correlation value (ρ>0.50) to prune even lightly redundant pairs of RFs couples. As an example, let A-B1 and A-B2 be two coupled RFs, sharing the single RF A, with B1, and B2 being single RFs too. Then, one of the two couples (either A-B1 or A-B2) was discarded. Finally, only 13 coupled RFs survived.

It is worth noting that, under the same ρ, first-order RFs were preferred with respect to second-order ones as well as lower distance GLCMs were preferred to higher ones (since it is known that GCLM distance is inversely proportional to their statistical significance).

Table S1. First-order RFs computed in the ROI, with related formulas and description

	1st order features	Formula	Description
1.	Mean	$M_{SUV} = \frac{1}{N_p}\sum_{i=1}^{N_p} X(i)$	The mean SUV value
2.	Standard deviation	$SD_{SUV} = \sqrt{\frac{1}{N_p}\sum_{i=1}^{N_p}(X(i) - \bar{X})^2}$	Dispersion of SUV values around their mean. A measure of visual contrast
3.	Median	$MED_{SUV} = \frac{X\left\lfloor\frac{N_p}{2}\right\rfloor + X\left\lceil\frac{N_p+1}{2}\right\rceil}{2}$	The "central" SUV value, with respect to whole SUV distribution
4.	Minimum	$MIN_{SUV} = min\,(X)$	The lowest SUV value
5.	Maximum	$MAX_{SUV} = max\,(X)$	The highest SUV value
6.	10th percentile	$10P_{SUV} = \frac{X\lfloor 0.1(N_p)\rfloor + X\lceil 0.1(N_p+1)\rceil}{2}$	The SUV value below which the 10% of the lowest SUV values can be found
7.	90th percentile	$90P_{SUV} = \frac{X\lfloor 0.9(N_p)\rfloor + X\lceil 0.9(N_p+1)\rceil}{2}$	The SUV value above which the 10% of the highest SUV values can be found
8.	Kurtosis	$K_{SUV} = \frac{\frac{1}{N_p}\sum_{i=1}^{N_p}(X(i) - \bar{X})^4}{(SD_{SUV})^4}$	Higher Kurtosis point out that most of SUV values are at the tails of distribution, lower values that are around the mean
9.	Skewness	$S_{SUV} = \frac{\frac{1}{N_p}\sum_{i=1}^{N_p}(X(i) - \bar{X})^3}{(SD_{SUV})^3}$	A measure of the asymmetry of SUV values around their mean
10.	Coefficient of variation	$CV_{SUV} = \frac{SD_{SUV}}{M_{SUV}}$	The level of dispersion of SUV values around their mean
11.	Entropy	$E_{SUV} = -\sum_{i=1}^{N_g} p(i)log_2(p(i))$	A measure of the randomness of SUV values
12.	Uniformity	$U_{SUV} = \sum_{i=1}^{N_g} p(i)^2$	A measure of the homogeneity of SUV values

Table S2. Second-order RFs computed in the ROI, with related formulas and description

	2st order features	Formula	Description
1.	Normalized homogeneity	$NH_{GLCM-d} = \sum_{i=1}^{N_g}\sum_{j=1}^{N_g}\frac{p(i,j)}{1+\left(\frac{i-j}{N_g}\right)^2}$	A measure of local SUV values homogeneity
2.	Angular second moment	$ASM_{GLCM-d} = \sum_{i=1}^{N_g}\sum_{j=1}^{N_g}\left(p(i,j)\right)^2$	A measure of the similarity between SUV values
3.	Entropy	$E_{GLCM-d} = -\sum_{i=1}^{N_g}\sum_{j=1}^{N_g} p(i,j)log_2\left(p(i,j)\right)$	A measure of randomness between neighbouring SUV values
4.	Difference entropy	$DE_{GLCM-d} = -\sum_{i=1}^{N_g} p_{x-y}(i)log_2\left(p_{x-y}(i)\right)$	A measure of randomness between neighbouring SUV value differences
5.	Contrast	$C_{GLCM-d} = \sum_{i=1}^{N_g}\sum_{j=1}^{N_g} p(i,j)(i-j)^2$	A measure of the local SUV variation, greater for distance voxels
6.	Joint maximum	$JM_{GLCM-d} = max\left(p(i,j)\right)$	The most predominant pair (at distance *d*) of SUV values
7.	Joint average	$JA_{GLCM-d} = \mu_x = \sum_{i=1}^{N_g}\sum_{j=1}^{N_g} p(i,j)i$	The joint averaged SUV value (*i*) distribution.
8.	Joint variance	$JV_{GLCM-d} = {\sigma_x}^2 = \sum_{i=1}^{N_g}\sum_{j=1}^{N_g} p(i,j)(i-\mu_x)^2$	The variance of the SUV values averaged by the their joint probability
9.	Correlation	$CORR_{GLCM-d} = \frac{\sum_{i=1}^{N_g}\sum_{j=1}^{N_g} p(i,j)ij - \mu_x\mu_y}{\sigma_x\sigma_y}$ where: $\mu_y = \sum_{i=1}^{N_g}\sum_{j=1}^{N_g} p(i,j)j$, $\sigma_y = \sqrt{\sum_{i=1}^{N_g}\sum_{j=1}^{N_g}\left(j-\mu_y\right)^2 p(i,j)}$	A measure of the linear dependency between SUV values and their respective voxels in the GLCM.
10.	Autocorrelation	$ACORR_{GLCM-d} = \sum_{i=1}^{N_g}\sum_{j=1}^{N_g} p(i,j)ij$	A measure of the magnitude of the fineness and coarseness of lesion's SUV texture.
11.	Inverse difference	$ID_{GLCM-d} = \sum_{i=1}^{N_g}\sum_{j=1}^{N_g}\frac{p(i,j)}{1+\lvert i-j\rvert}$	A measure of lesion's SUV values local homogeneity
12.	Cluster tendency	$CT_{GLCM-d} = \sum_{i=1}^{N_g}\sum_{j=1}^{N_g} p(i,j)\left(i+j-\mu_x-\mu_y\right)^2$	A measure of whether the SUV of lesion contains meaningful non-random structures

Table S3: Main performance of the coupled features (**$NH_{GLCM-\{1,2\}}$, $E_{GLCM-\{3,4\}}$**)

Second-order features	AUC	p-value	SN	SP	YI
**(NH_{GLCM-2}, E_{GLCM-4})**	**0.94 (95% CI, 0.76-0.99)**	**0.0001**	**92%**	**85%**	**0.77**
(NH_{GLCM-2}, E_{GLCM-3})	0.90 (95% CI, 0.65-0.98)	0.0005	92%	85%	0.77
(NH_{GLCM-1}, E_{GLCM-4})	0.94 (95% CI, 0.65-0.99)	0.0007	85%	92%	0.77
(NH_{GLCM-1}, E_{GLCM-3})	0.91 (95% CI, 0.66-0.99)	0.0020	92%	85%	0.77

Table S4: Resuming the main performance parameters of models A and B on training (HS) and validation (BS) cohorts. ρ=Pearson correlation coefficient, SN=sensitivity, SP=specificity, ACC=accuracy, PPV=positive predictive value, NPV=negative predictive value, I=informedness, AUC=area under ROC curve.

Model	Cohort	AUC	p-value	ρ	SN	SP	ACC	PPV	NPV	I
A	Training on HS	0.94 (95% CI, 0.74-0.99)	2.4e-4	0.03	0.92	0.85	0.88	0.85	0.92	0.76
	Validation on BS	0.90 (95% CI, 0.62-1.00)	3.5e-3	-0.19	0.88	0.83	0.85	0.70	0.94	0.71
B	Training on BS	0.87 (95% CI, 0.57-0.99)	3.5e-3	0.19	0.63	0.89	0.81	0.71	0.84	0.51
	Validation on HS	0.92 (95% CI, 0.75-0.99)	2.4e-4	0.03	0.83	0.92	0.88	0.91	0.86	0.76

Table S5: Resuming AUC values for training and validation of model C referring to all 300 runs, to all non-overfitting models, and to the winning models found. N=number of runs, Std=standard deviation, Med=median, IQR=interquartile range.

**Cohort**	**Models**	**N**	**Mean**	**Std**	**Med**	**IQR**	**Min**	**Max**	**p-value range**
Training	All 300	300	0.91	0.03	0.91	0.05	0.84	0.98	3.4e-6 – 9.8e-4
	Non-overfitting	161	0.93	0.02	0.93	0.03	0.88	0.98	3.4e-6 – 2.6e-4
	Winning	99	0.93	0.02	0.93	0.03	0.88	0.98	3.5e-6 – 2.6e-4
Test	All 300	300	0.89	0.08	0.90	0.11	0.57	1	1.0e-4 – 6.7e-4
	Non-overfitting	126	0.86	0.03	0.86	0.06	0.80	0.91	3.1e-3 – 4.8e-2
	Winning	79	0.87	0.03	0.87	0.05	0.80	0.91	3.1e-3 – 4.8e-2
